# Prevalence of Misrepresentation of Nonphysician Clinicians at Dermatology Clinics

**DOI:** 10.7759/cureus.18793

**Published:** 2021-10-14

**Authors:** Andrew Creadore, Sheena Desai, Sara J Li, Karen Lee, Eric Xia, Ai-Tram N Bui, Camila Villa-Ruiz, Kelly Lo, Arash Mostaghimi

**Affiliations:** 1 Department of Dermatology, Brigham & Women's Hospital, Harvard Medical School, Boston, USA; 2 Department of Dermatology, Boston University School of Medicine, Boston, USA; 3 Department of Dermatology, Tufts University School of Medicine, Boston, USA; 4 Department of Dermatology, Harvard Medical School, Boston, USA; 5 Department of Dermatology, Ponce Health Sciences University, Ponce, PRI; 6 Department of Dermatology, Brigham & Women’s Hospital, Harvard Medical School, Boston, USA

**Keywords:** nonphysician clinicians, dermatology board certification, mid-level providers, advanced practice professionals, physician assistant, nurse practitioner

## Abstract

Introduction: To evaluate the use of inaccurate terminology used by dermatology practices to describe the training and qualifications of their nonphysician clinicians (NPCs) when new patients are booking appointments.

Methods: Clinics were randomly selected and called to determine the first available appointment for a new patient with a new and changing mole. If the receptionist confirmed the first-offered appointment was with an NPC, the encounter was included in this study. If receptionists used inaccurate terminology to describe the NPCs and their qualifications, this instance was recorded along with the specific language that they used.

Results: A total of 344 unique dermatology clinics were contacted on February 27, 2020, in 25 states. Phone calls at 128 clinics (37.2%) met our inclusion criterion. Inaccurate language was used to describe NPCs at 23 (18%) unique clinic locations across 12 states, with “dermatologist,” “doctor,” “physician,” and “board-certified” being used to describe NPCs as the most common inaccurate terms.

Conclusion: These findings demonstrate that front office staff at dermatology clinics use inaccurate and potentially misleading terminology to refer to NPCs working in their clinics. While we cannot establish whether this is intentional or due to a lack of training, additional focus should be placed on accurately representing provider qualifications to patients.

## Introduction

The use of nonphysician clinicians (NPCs), defined here as physician assistants and nurse practitioners, at dermatology clinics has increased over time [1,2]. Although these providers are an essential part of the dermatology workforce, there are concerns regarding the accurate reflection of the training and qualifications of NPCs by practices that employ them [2,3]. We evaluated the use of inaccurate terminology used by dermatology practices to describe the qualifications of their NPCs when new patients are booking appointments.

## Materials and methods

This study is an extension of a prior secret shopper study of dermatology appointment access at clinics with and without private equity (PE) ownership that provided adult medical dermatology services [4]. Clinics were randomly selected using a specific protocol described previously and called using a standardized script to determine the first available appointment for a new patient with a new and changing mole [4]. After learning the date of the first available appointment, we inquired what the type of provider for this appointment would be. If the receptionist confirmed the first-offered appointment was with an NPC, the encounter was included in this study.

Per protocol, if an appointment with an NPC was offered, the receptionist was next asked when the next appointment was available with a physician dermatologist instead of an NPC [4]. If, in response to this inquiry, the receptionist used inaccurate terminology to describe the NPCs and their qualifications, we recorded this instance along with the specific language that they used.

## Results

A total of 344 unique dermatology clinics were contacted on February 27, 2020, in 25 states (Table 1) [4]. Phone calls at 128 clinics (37.2%) met our inclusion criterion for data collection. Inaccurate language was used to describe NPCs at 23 (18%) unique clinic locations across 12 states, with “dermatologist,” “doctor,” “physician,” and “board-certified” being used to describe NPCs as the most common inaccurate terms (Table 2).

**Table 1:** Percentage of contacted clinics meeting inclusion criteria and using inaccurate terminology *Geographic regions determined based on previously published data [1]

Clinic Category	Number of Clinics Contacted	Inclusion Criteria Met	Inaccurate Terminology Used
Total	344	128/344 (37.2%)	23/128 (18%)
Northeast*	92	29/92 (31.5%)	3/29 (10.3%)
South*	195	65/195 (33.3%)	9/65 (13.8%)
Midwest*	69	28/69 (40.6%)	3/28 (10.7%)
West*	106	25/106 (23.6%)	8/25 (32%)
Clinics with PE ownership	111	48/111 (43.2%)	10/48 (20.8%)
Clinics without PE ownership	233	80/233 (34.3%)	13/80 (16.3%)

**Table 2:** Prevalence of specific inaccurate terminology used to describe NPCs at dermatology clinics

Prevalence of Specific Inaccurate Terminology	
Terminology Used	N (%)
Dermatologist	9 (7%)
Doctor	5 (4%)
Physician (not followed by assistant)	4 (3%)
Board certified	4 (3%)
They do the same thing as doctors	2 (1%)
Specialize in dermatology	1 (1%)
Sample of Representative Statements From Calls	
“We have three mid-levels and two physicians. They are all dermatologists. They all specialize in dermatology.”	
“Physician assistants are all capable of diagnosing dermatology diseases and are all board certified in dermatology.”	
“All our physician assistants specialize in dermatology, and they do their own diagnosing and surgeries.”	
“Just so you know, the physician assistant is a dermatologist too; they are dermatology certified.”	
“We don't have doctors, but all our staff are certified in dermatology.”	
“Our doctor there only works Tuesdays and Wednesdays.”	
“They can treat, diagnose, and biopsy just like a doctor can.”	
“They are physician assistants; they are doctors of dermatology.”	
“Well, she is a dermatologist.”	
“They’re all dermatologists.”	
“The physician assistant is a physician.”	

## Discussion

Although most clinics did not misrepresent the qualifications of their NPCs, clinics in multiple states, irrespective of the ownership model, similarly used a variety of terms that are objectively inaccurate. The American Board of Dermatology defines a dermatologist as a physician whose certification necessitates, among other requirements, an MD or DO degree from a recognized school of medicine or osteopathy and multiple years of training in both general medicine and, specifically, disease of the skin, hair, and nails [5].

Although NPCs who are appropriately supervised play an important role in the care of dermatology patients in the United States, training for NPCs is not standardized and has no guarantee to include dermatology-specific training [2]. Statements contradicting this or attempting to convince potential patients that outcomes under both provider types will be equivalent are inaccurate and diminish the importance and necessity of the additional training required of physicians [2-4].

This study has several important limitations. We only have data from 128 clinics, and studies on a larger scale or presenting varying clinical scenarios may have different results. Additionally, our study was unable to distinguish between intentional misrepresentation of NPCs and other potential causes of these findings, such as the knowledge gaps of office staff or misinformation from administrators. Further, this study excluded academic and federal clinics, as well as clinics providing only pathology, surgical, or cosmetic services [4]. These clinics, which also employ NPCs, represent a gap in knowledge that may or may not result in distinct findings.

## Conclusions

Although we are unable to ascertain the intentionality of misrepresentation, our findings suggest a need to focus on accurate and accessible language to describe NPCs employed by dermatology clinics. In order to prevent misrepresentation between various provider types, such clinics should consistently use transparent and clearly defined titles, qualifications, and levels of training. Further, clinics should educate staff members who take phone calls to ensure that appropriate, standardized language is consistently used. Doing so will not only maintain the integrity of the term “dermatologist” but also enable patients to make informed decisions about their providers when seeking care.
